# Saltatory conduction in unmyelinated axons: clustering of Na^+^ channels on lipid rafts enables micro-saltatory conduction in C-fibers

**DOI:** 10.3389/fnana.2014.00109

**Published:** 2014-10-13

**Authors:** Ali Neishabouri, A. Aldo Faisal

**Affiliations:** ^1^Brain and Behaviour Lab, Department of Computing, Imperial College LondonLondon, UK; ^2^Brain and Behaviour Lab, Department of Bioengineering, Imperial College LondonLondon, UK; ^3^Faculty of Medicine, MRC Clinical Sciences CentreLondon, UK

**Keywords:** C-fiber, Nav1.8, Hodgkin-Huxley, action potential, axon, lipid raft, saltatory conduction

## Abstract

The action potential (AP), the fundamental signal of the nervous system, is carried by two types of axons: unmyelinated and myelinated fibers. In the former the action potential propagates continuously along the axon as established in large-diameter fibers. In the latter axons the AP jumps along the nodes of Ranvier—discrete, anatomically specialized regions which contain very high densities of sodium ion (Na^+^) channels. Therefore, saltatory conduction is thought as the hallmark of myelinated axons, which enables faster and more reliable propagation of signals than in unmyelinated axons of same outer diameter. Recent molecular anatomy showed that in C-fibers, the very thin (0.1 μm diameter) axons of the peripheral nervous system, Nav1.8 channels are clustered together on lipid rafts that float in the cell membrane. This localized concentration of Na^+^ channels resembles in structure the ion channel organization at the nodes of Ranvier, yet it is currently unknown whether this translates into an equivalent phenomenon of saltatory conduction or related-functional benefits and efficiencies. Therefore, we modeled biophysically realistic unmyelinated axons with both conventional and lipid-raft based organization of Na^+^ channels. We find that APs are reliably conducted in a micro-saltatory fashion along lipid rafts. Comparing APs in unmyelinated fibers with and without lipid rafts did not reveal any significant difference in either the metabolic cost or AP propagation velocity. By investigating the efficiency of AP propagation over Nav1.8 channels, we find however that the specific inactivation properties of these channels significantly increase the metabolic cost of signaling in C-fibers.

## 1. Introduction

Propagation of action potentials (AP) in axons relies on the concerted action of membrane-spanning selectively permeable ion channels (Hodgkin and Huxley, 1952). Myelinated axons feature a highly structured distribution of voltage-gated ion channels, with a characteristic clustering of Na^+^ channels at the nodes of Ranvier. Saltatory conduction (Huxley and Stämpfli, 1949; Fitzhugh, 1962) in myelinated axons refers to the rapid propagation of the electrical waveform from each node to the next (the AP seems to jump between nodes). This mode of conduction allows faster (Rushton, 1951; Waxman and Bennett, 1972) and more reliable (Kuriscak et al., 2002) propagation of signals than unmyelinated axons. In contrast, only unmyelinated axons, which are generally feature uniformly distributed ion channels (Black et al., 1981), are found at diameters approaching the physical limits to axon diameter (Waxman and Bennett, 1972) (*d*) at 0.1 μm (Faisal et al., 2005), thus making the high connection densities of mammalian cortex possible.

The number of ion channels on the surface of neurons' membrane is usually thought to be large enough to justify combining the individual channel conductances into a continuous measure of overall conductivity (Dayan and Abbott, 2001), as originally done by Hodgkin and Huxley (1952). However in the case of thin axons, the number of ion channels may be too small for these approximations to be valid. Faisal and Laughlin (2007) showed that in order to accurately model thin axons, the behavior of individual ion channels needs to be taken into account. Channel noise in very thin axons has a large effect, limiting the miniaturization of fibers by imposing a lower diameter on axons at 0.1 μm (Faisal et al., 2005). The conceptual transition from conductivity (per surface area) to density of channels, with each channel having only two possible conductance value corresponding to its open and closed states, involves investigating the effects of possible non-uniformities in the distribution of ion channels across the membrane.

Based on observations from the neonatal rat optic nerve, Waxman et al. (1989) hypothesized that action potentials could be propagated along thin (*d* ≈ 0.2 μm) axons by “jumping” between individual Na^+^ channels placed a few microns apart. This postulated mode of propagation would be the analog of saltatory conduction in myelinated axons (Huxley and Stämpfli, 1949) and was termed micro-saltatory conduction. Faisal and Laughlin (2007) showed that probabilistic gating of ion channels due to thermodynamic fluctuations, or channel noise (reviewed by White et al., 2000) makes micro-saltatory conduction between individual Na^+^ channels impossible. This is because the very low diameters required for individual Na^+^ channels to have a measurable effect on the membrane potential make the axon overly sensitive to stochastic opening of Na^+^ channels, resulting in an excessive spontaneous AP rate.

In C-fibers, Pristerà et al. (2012) have recently discovered that NaV1.8, the voltage-gated Na^+^ channels of these 0.1 μm diameter unmyelinated axons (Sangameswaran et al., 1996; Baker, 2005), are packed tightly together on lipid rafts. Lipid rafts are “dynamic, nanoscale, sterolsphingolipids enriched, ordered assemblies of proteins and lipids” (Pike, 2006; Coskun and Simons, 2010; Simons and Gerl, 2010). They play a role in organizing the cell membrane, and act as hubs for functional localization of proteins (Pristerà et al., 2012). They also intervene in trafficking, clustering and electrophysiological properties of ion channels, and have an effect on cell excitability (reviewed by Pristerá and Okuse, 2012). They are typically 0.1–0.3 μm long (Personal communication, Amber Finn and Kenji Okuse, 2011), and placed ~5–10 μm apart (Pristerà et al., 2012). Disassociating lipid rafts and Nav1.8 channels in DRG neurons is correlated with impaired neuronal excitability (Pristerà et al., 2012).

C-fibers are very thin unmyelinated peripheral axons responsible for transmitting nociceptive pain sensations (Lawson, 2002). A variety of Na^+^ channels are found on the membrane of C-fibers, including TTX-sensitive Nav1.6 (Black et al., 2002) and Nav1.7 (Black et al., 2012) channels. Voltage clamp experiments have shown that these TTX-sensitive channels are involved in amplifying subthreshold depolarizations, and are active during APs (Vasylyev and Waxman, 2012). The slow-activating, slow-inactivating Nav1.8 channels play a crucial part in the generation and propagation of APs in these fibers (Akopian et al., 1999; Renganathan et al., 2001; Lai et al., 2003). As a result, these TTX-resistant channels are of particular interest for treating neuropathic pain symptoms (reviewed by Scholz and Woolf, 2002). The clustering of Na^+^ channels on lipid rafts resembles the structure of nodes of Ranvier in myelinated fibers, and may permit micro-saltatory conduction in those thin axons. Here, we investigate whether this mode of propagation is indeed possible, and its potential benefits in terms of basic constraints faced by neural fibers.

## 2. Materials and methods

We investigated the effects of the lipid-raft clustering of Na^+^ channels on the function of neural fibers, using both deterministic and stochastic simulations. In stochastic simulations, the changes of conformations of ion channels were individually modeled (Faisal, 2012). Simulations were based on biophysical data from Baker (2005). Computations were carried out using the Modigliani stochastic simulator (Faisal et al., 2002, 2005), on a Linux PC using an Intel core i7 processor with the binomial algorithm, chosen because it allows accurate simulations that are less computationally intensive than the Gillespie algorithm (Faisal, 2010). Membrane capacitance was set to 0.81 μF cm^−2^, axial resistance to 70 Ω cm, and the membrane leak conductance was 0.14 mS cm^−2^. Leak reversal potential, Na^+^ reversal potential and K^+^ reversal potentials were, in order, −61.14, 79.6, and −85 mV.

Our C-fiber model axon contains only two types of voltage gated ion channels. We use a model of TTX-resistant Na^+^ channels (NaV1.8) based on physiological data from Baker (2005). The instantaneous Na^+^ conductance in the model is given by *g*_Na^+^_ = *g*_Na^+^_ × *m*^3^*h* where *g*_Na^+^_ = 1.25 mS cm^−2^. *m* and *h* follow the classical Hodgkin and Huxley (1952) dynamics, with rates α_*m*_ = 3.83/(1 + *exp*((*V_m_* + 2.58)/ − 11.47)), β*_m_* = 6.894/(1 + *exp*((*V_m_* + 61.2)/19.8)), α*_h_* = 0.013536 × *exp*(−(*V_m_* + 105)/46.33) and β*_h_* = 0.61714/(1 + *exp*((*V_m_* − 21.8)/ − 11.998)). This transforms into the 8-state Na^+^ channel model for stochastic simulations.

**Figure 1:**
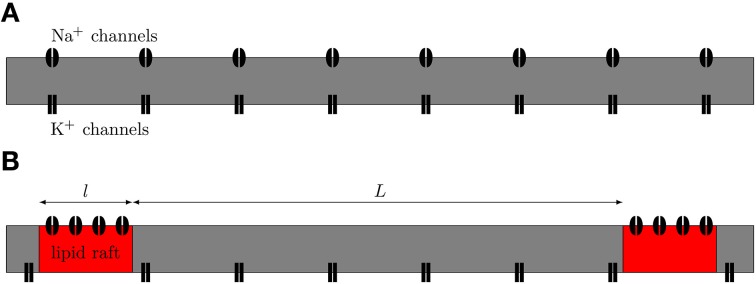
**Schematic view of axonal models. (A)** The null-hypothesis axon. Both Na^+^ and K^+^ channels are uniformly diffused along the axon. **(B)** Axon with Na^+^ channels clustered together on lipid rafts. We model this axon by placing a compartment containing a high density of Na^+^ channels at regular distances in between compartments containing only K^+^ channels.

We also used the model for fast K^+^ channels given by Baker (2005). The instantaneous K^+^ conductance in the model is given by *g*_K^+^_ = *g*_K^+^_ × *n*^4^ where *g*_K^+^_ = 0.17 mS cm^−2^. and the kinetic rates for *n* are given by α*_n_* = 0.00798(*V_m_* + 72.2)/(1 − *exp*((− 72.2 − *V_m_*)/1.1)) and β*_n_* = 0.0142(− 55 − *V_m_*)/(1 − *exp*((*V_m_* + 55)/10.5)). This transforms into a 5-state channel for stochastic simulation (for details, see Faisal et al., 2002),

We simulated both uniformly distributed channels and channels clustered on lipid rafts placed regularly along the 0.1 μm diameter C-fiber axon (see Figure 1). For the uniformly distributed Na^+^ channels axon model, we use a single Na^+^ channel conductance of 20 pS, which translates into a density of 56.25 μm^−2^, and a single K^+^ channel conductance of 17 pS, which translates into a density of 10 μm^−2^. Single channel conductance values are putative (based on typical values for ion channels). For the clustered Na^+^ channel model, we kept the density of K^+^ channels constant in the region between lipid rafts. For comparisons between the axon with uniformly distributed Na^+^ channels and the one with lipid rafts, the overall Na^+^ density was kept constant (900 μm^−2^ for 0.2 μm long rafts placed 3 μm apart). We also simulate different cluster configurations (length, distance) based on previous work (Zeng and Tang, 2009) although the results from those can not be directly compared to the uniform axon. On the lipid rafts there are no K^+^ channels. For all lipid raft configurations i.e., all values of raft length *l* and distance between rafts *L*, we simulated an axon long enough to contain 100 lipid rafts. At each trial, we injected a small current step twice. The first evoked AP was only used to ensure the ion channels were properly initialized. We only used data from the second AP of each trial.

The width of each AP is measured between the half-width points. The metabolic cost of AP propagation is usually defined as the amount of ATP molecules necessary to reverse the Na^+^ current by Na^+^-K^+^-ATPase (Alle et al., 2009; Sengupta et al., 2010). However, we chose to keep this measure in terms of Na^+^ charge and not convert it into a measure in terms of the amount of ATP molecules, because Na^+^ charge is given directly by the amount of current crossing the membrane, and does not require additional assumptions on how the Na^+^ charge is reversed.

## 3. Results

**Figure 2:**
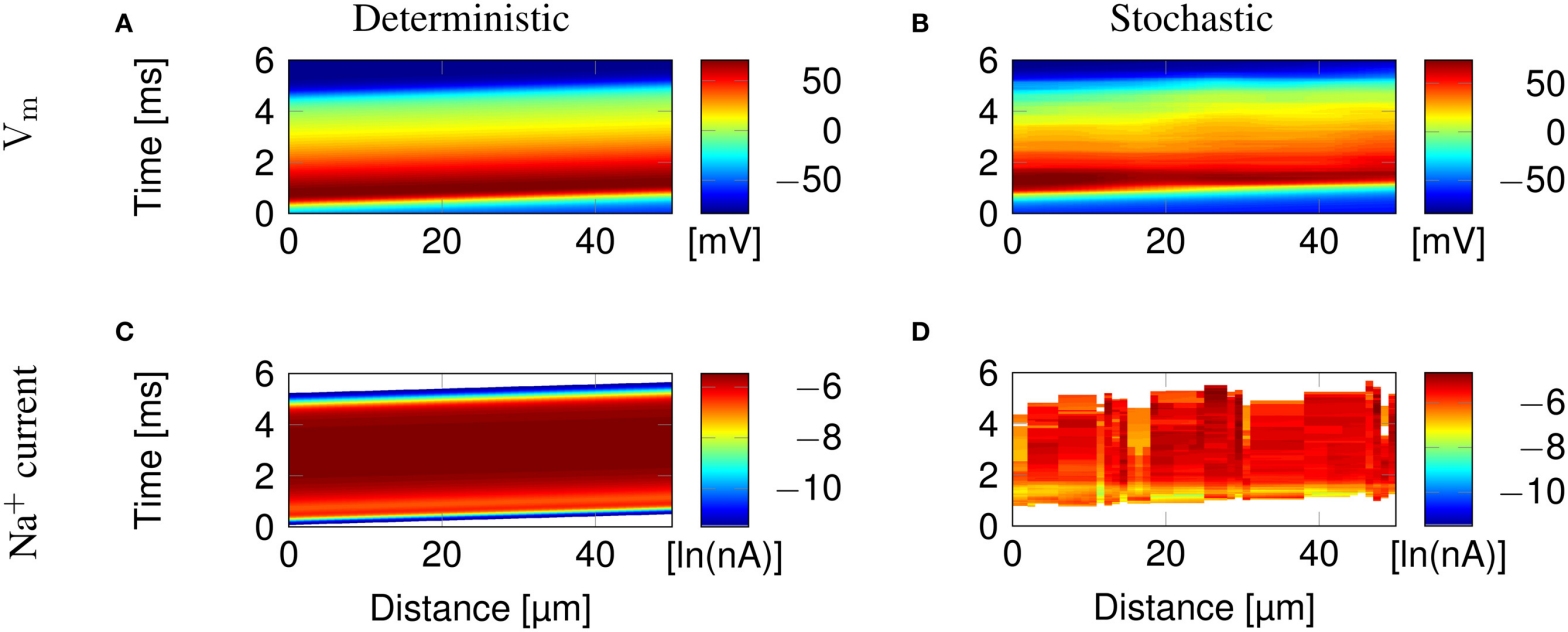
**Action potential propagation in a 0.1 μm diameter C-fiber axon with uniformly distributed Na^+^ channels. (A)** The membrane potential and **(C)** Na^+^ current in a deterministic simulation. **(B)** The membrane potential and **(D)** Na^+^ current in a single trial of stochastic simulations. White areas signify no current flow.

Figure 2 illustrates the propagation of an action potential in a C-fiber axon with uniformly distributed Na^+^ channels, using both deterministic and stochastic simulations. In deterministic simulations, the AP waveform is kept constant while propagating through the axon. The activation profile of Na^+^ channels, and hence the Na^+^ current, is also the same at all points along the axon, as we expect.

In stochastic simulations using discrete, stochastic ion channels, there is considerable variability in the Na^+^ current crossing the axon, as shown by the profile of Na^+^ current Figure 2D. In addition, the stochastic opening of each discrete channel has a minimum current flow determined by the single channel conductance, that is larger than the minimum conductance allowed in the deterministic model. This is visible in the absence of blue bands of low Na^+^ current in Figure 2D, although they are present at the beginning and end of the AP in Figure 2C.

### 3.1. Microsaltatory conduction along lipid rafts

**Figure 3:**
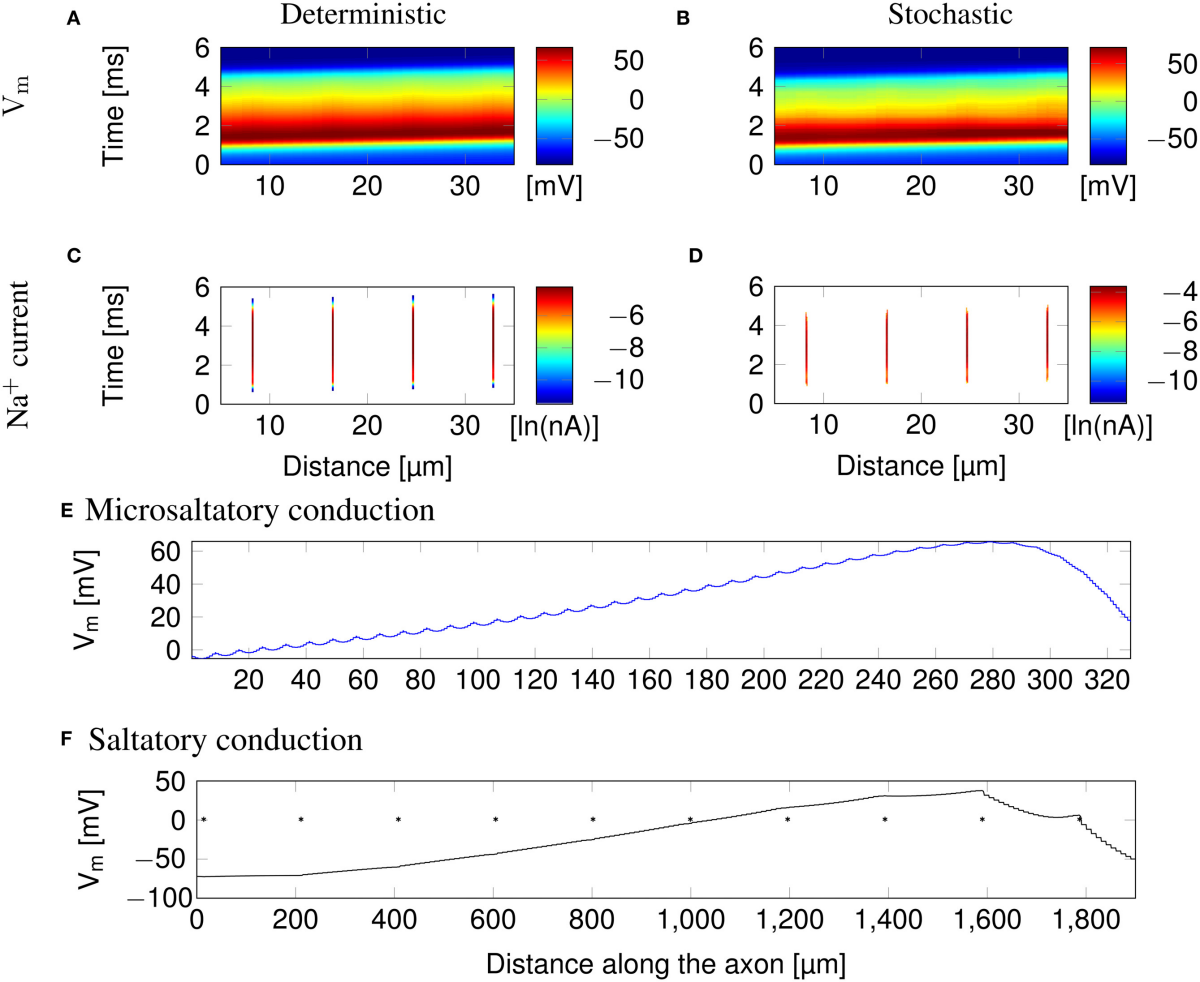
**Microsaltatory conduction in a 0.1 μm diameter C-fiber axon with clustered Na^+^ channels (*l* = 0.2 μm, *L* = 8 μm). (A)** The membrane potential and **(C)** Na^+^ current in a deterministic simulation. **(B)** The membrane potential and **(D)** Na^+^ current in a single trial of stochastic simulations. In **(C,D)** the lines of Na^+^ current are very fine due to the short length of lipid rafts. **(E)** Profile of the AP waveform propagating in a C-fiber axon. The bumps in the waveform correspond to the placement of lipid rafts. **(F)** Profile of the AP waveform in a model of mammalian myelinated axon (using parameters from McIntyre et al., 2002). The AP “jumps” between nodes of Ranvier, denoted by ^*^, but the amplitude is not heavily reduced over the internode.

Clustering Na^+^ channels on putative lipid rafts still allows AP conduction (Figure 3). In both deterministic (Figures 3A,C) and stochastic (Figures 3B,D) simulations, the AP is sustained by the Na^+^ current in lipid rafts alone. Plotting the profile of the AP waveform (Figure 3E) shows bumps in the waveform, corresponding to the placement of Na^+^ channel lipid rafts.

The height of the AP is only slightly lower outside of lipid rafts. This is because *L* is much lower than the axon's length constant λ. Therefore, the membrane potential over the inter-raft region is roughly constant, and equal to that over lipid rafts. Note that in myelinated axons, the amplitude of APs over the internodal regions is also not much lower than the amplitude in nodes of Ranvier (Bakiri et al., 2011). This is also confirmed by our simulations of a mammalian myelinated axon model (see Figure 3F), based on data from McIntyre et al. (2002).

**Figure 4:**
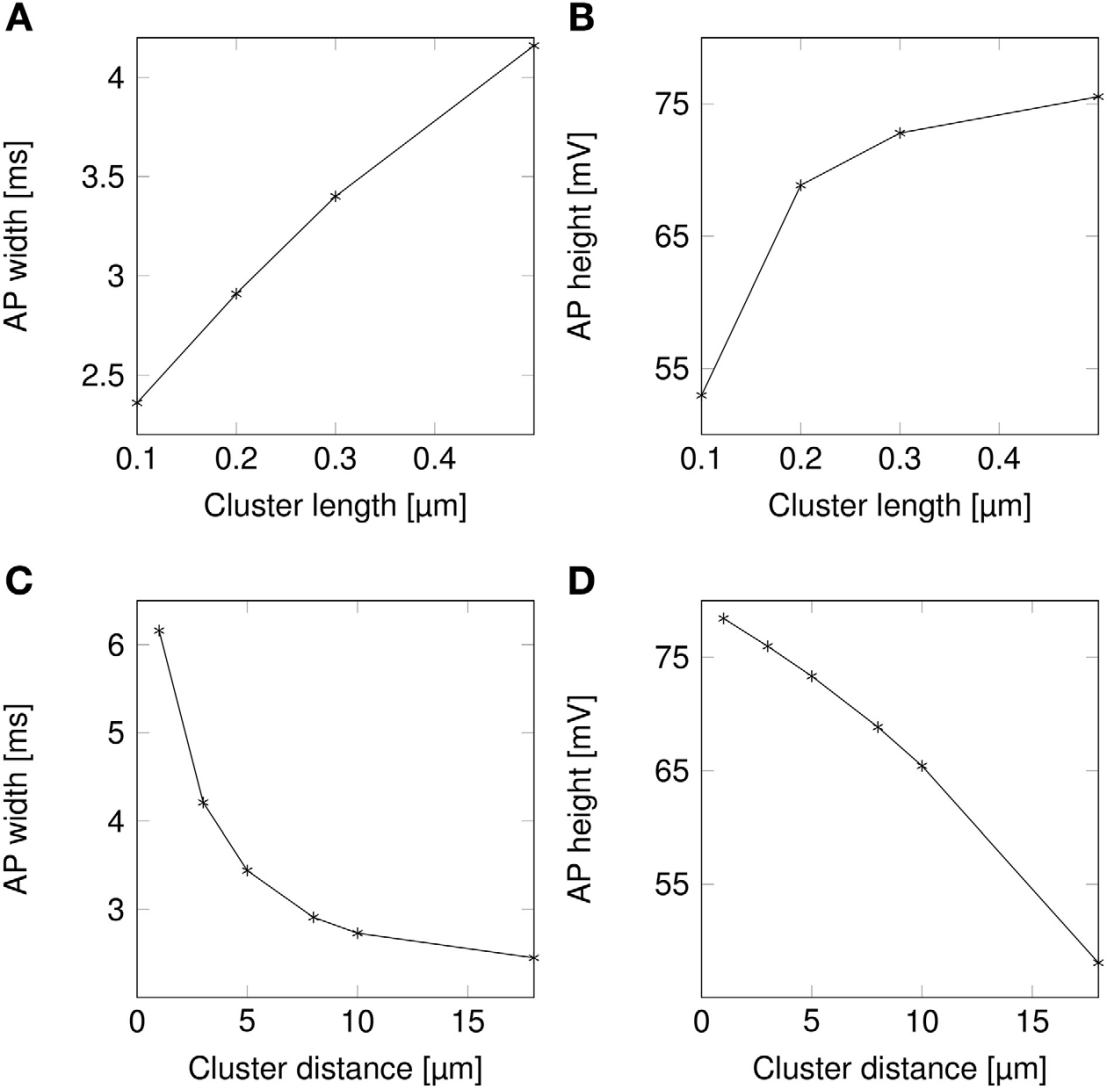
**Action potential height and width as functions of channel distributions**. When varying the length of lipid rafts, the inter-raft distance was set to 8 μm. While varying the inter-raft distance, the lipid raft length was set to 0.2 μm. **(A)** Action potential width (measured at half-peak value) in a model axon with clustered Na^+^ channels as a function of raft length. **(B)** Action potential peak in a model axon with clustered Na^+^ channels as a function of raft length. **(C)** Action potential width (measured at half-peak value) in a model axon with clustered Na^+^ channels as a function of distance between lipid rafts. **(D)** Action potential peak in a model axon with clustered Na^+^ channels as a function of distance between rafts.

AP waveforms are slightly wider over lipid rafts in both deterministic (Figure 3A) and stochastic (Figure 3B) simulations. This effect is due to the reopening of Na^+^ channels in the repolarizing phase of the AP (**Figure 8**). We investigated the influence of the length (*l*) of rafts, and the distance between them (*L*) on the shape of action potential. The results are plotted in Figure 4. Both AP width and height seem affected by the size and placement of lipid rafts. Longer rafts increase the width of APs almost linearly (Figure 4A). The greater number of Na^+^ channels in longer lipid rafts also pushes the peak of APs toward the Na^+^ reversal potential, increasing it from ~55 to 75 mV (Figure 4B). Increasing the distance between lipid rafts, on the other hand, shortens the width of APs. With 0.2 μm long rafts 10 μm apart, the AP width is halved compared to placing the rafts only 1 μm apart (Figure 4C). The height of APs is also reduced by furthering lipid rafts, this time in an almost linear fashion (Figure 4D) which is expected since the change is no longer limited by the Na^+^ reversal potential.

Changes in the height and width of APs are expected because of the changes in the overall Na^+^ channel density that is caused by changes in the size or placement of lipid rafts, and not the clustering of Na^+^ channels *per se*. We treat this question in Section 3.5.

### 3.2. Change in shapes of APs results in lower metabolic costs

**Figure 5:**
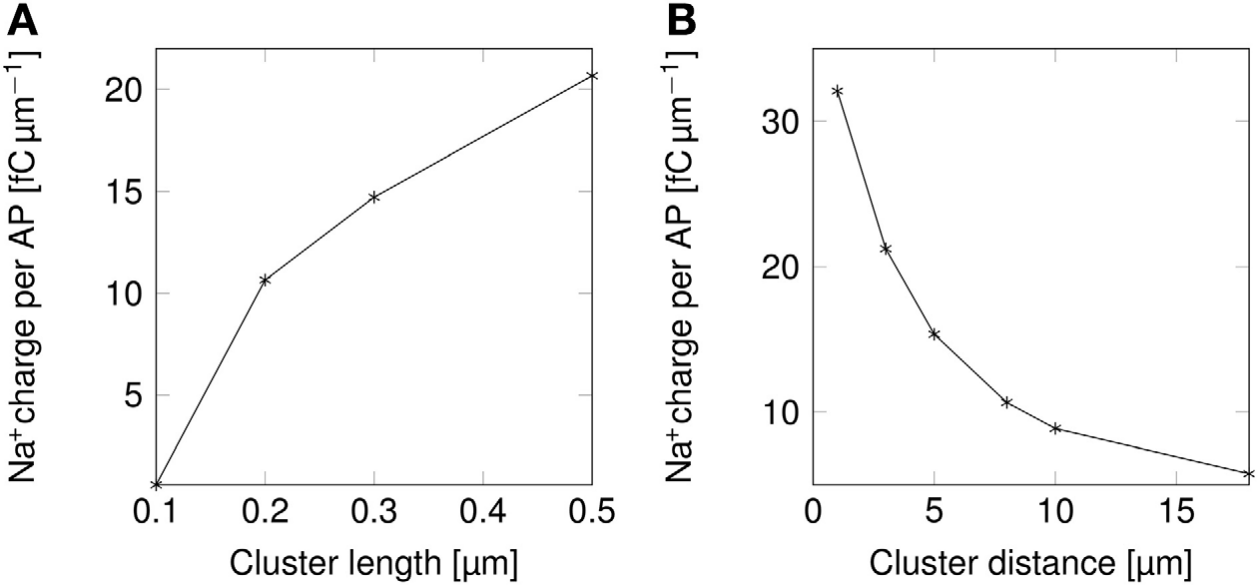
**Metabolic cost of action potentials as functions of lipid raft configuration in a 0.1 μm diameter C-fiber axon. (A)** Metabolic cost of action potentials in a model axon with clustered Na^+^ channels for varying lipid rafts sizes. Distance between rafts was set to 8 μm. **(B)** Metabolic cost of action potentials in a model axon with clustered Na^+^ channels for varying inter-raft distances. Lipid raft length was set to 0.2 μm.

The change in the shape of APs directly results into changes in their metabolic cost (Figure 5). Shortening lipid rafts lowers the amount of Na^+^ charge crossing the membrane and thus the cost in ATP associated with pumping Na^+^ ions back out of the cell (Figure 5A). Increasing the distance between rafts also reduces the metabolic cost. The profile of the variation in metabolic cost closely follows that of change in AP width, suggesting that width, rather than height, determines the metabolic cost of firing APs (Figures 4A,C, 5).

**Figure 6:**
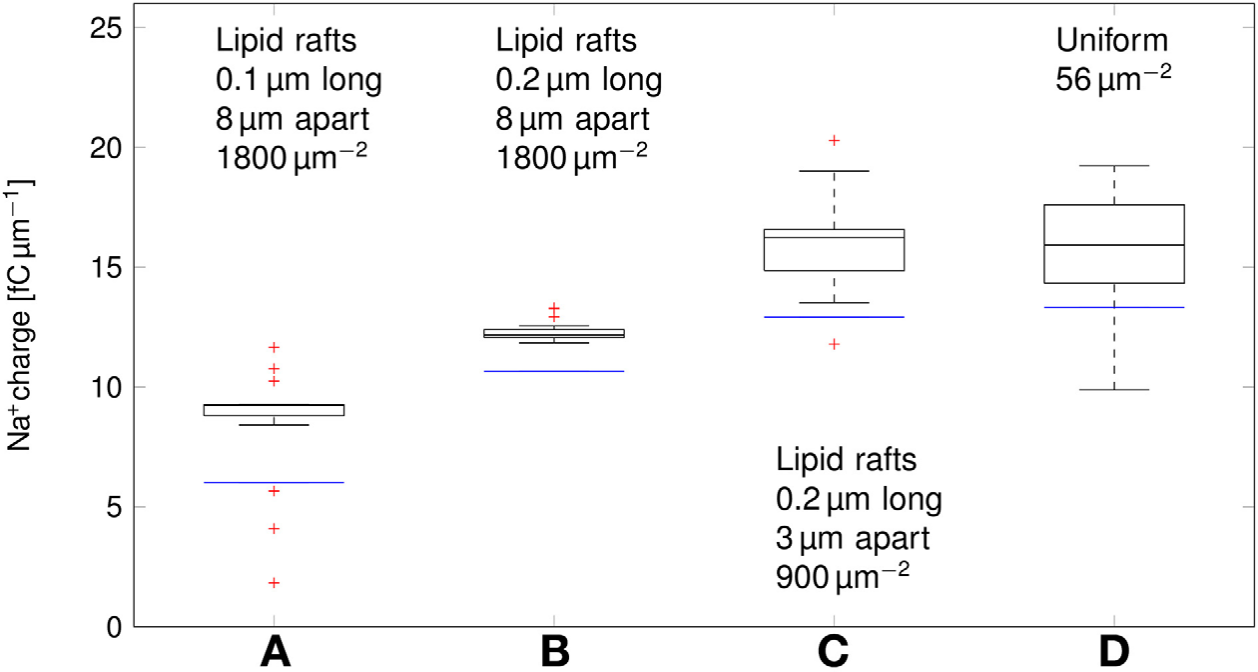
**Metabolic cost of action potentials for different channel distributions in a 0.1 μm diameter C-fiber axon**. Data was obtained using both deterministic (blue lines) and stochastic (boxes) simulations. **(A)** *l* = 0.1 μm, *L* = 8 μm, optimal values given by Zeng and Tang (2009), Na^+^ channel density 1800 μm^−2^, **(B)** *l* = 0.2 μm, *L* = 8 μm, Na^+^ channel density 1800 μm^−2^, **(C)** *l* = 0.2 μm, *L* = 3 μm, Na^+^ channel density 900 μm^−2^, based on data on lipid raft placement given by Pristerà et al. (2012) and personal communication with Amber Finn, **(D)** Uniform distribution. On each box, the central mark is the median, the edges of the box are the 25th and 75th percentiles, the whiskers extend to the most extreme data points not considered outliers, and outliers are plotted individually.

We can now compare the metabolic cost of APs in axons with clustered Na^+^ channels to the cost in axons where Na^+^ channels are uniformly distributed (Figure 6). Experimental results (Personal communication, Amber Finn) suggest *l*= 0.1–0.3 μm long lipid rafts placed *L*≈3 μm apart. The overall density of Na^+^ channels is kept constant by assuming a density of 900 μm^−2^ in the lipid rafts. In our simulation, this does not result in a significant change of metabolic cost. The metabolic cost for propagating APs over axons with both clustered and uniformly distributed Na^+^ channels is ~13 fC μm^−1^ for the 0.1 μm diameter axon in deterministic simulations. In stochastic simulations, the opening of a channel means that a conductance equal to that of the single channel is added to the membrane. This minimum current due to the discrete nature of ion channel conductance has an impact on the metabolic cost of APs. Stochastic simulations yield a median value of 16 fC μm^−1^ per AP in axon with clustered or uniformly distributed channels.

Increasing *L*, and partially compensating by increasing the density of Na^+^ channels in lipid rafts lowers the Na^+^ charge crossing the membrane: 0.2 μm long rafts placed 8 μm apart (Zeng and Tang, 2009) are more metabolically efficient than uniformly placed Na^+^ channels (~11 fC cm^−1^ in deterministic simulations). Shortening the lipid rafts to 0.1 μm reduces the Na^+^ charge even further, while maintaining the axon's capacity to propagate APs.

### 3.3. Propagation velocity over clustered Na^+^ channels

Due to their very small diameter, it is extremely difficult to obtain intracellular data from C-fibers, and therefore we can only estimate the propagation velocity in these fibers using extracellular recordings (Tigerholm et al., 2014). These estimations can not be reliably linked to axonal diameter. C-fiber axons are known for their very low conduction velocities. The conduction velocity is estimated to be 69cm s^−1^ for a 0.25 μm diameter axon (Tigerholm et al., 2014).

**Figure 7:**
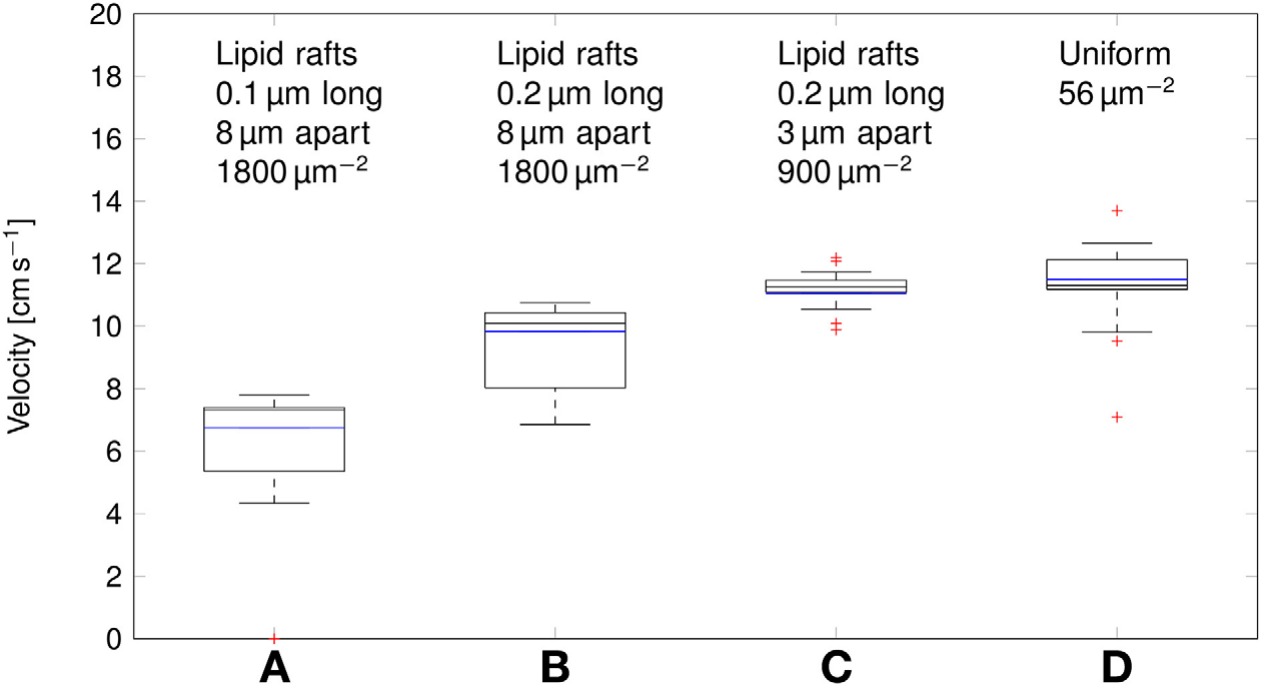
**Propagation velocity of action potentials for different channel distributions in a 0.1 μm diameter C-fiber axon**. Data was obtained using both deterministic (blue lines) and stochastic (boxes) simulations. **(A)** *l* = 0.1 μm, *L* = 8 μm, optimal values given by Zeng and Tang (2009), Na^+^ channel density 1800 μm^−2^, Note that in stochastic simulations some APs failed to propagate. **(B)** *l* = 0.2 μm, *L* = 8 μm, Na^+^ channel density 1800 μm^−2^, **(C)** *l* = 0.2 μm, *L* = 3 μm, Na^+^ channel density 900 μm^−2^, based on data on lipid raft placement given by Pristerà et al. (2012) and personal communication with Amber Finn. **(D)** Uniform distribution. On each box, the central mark is the median, the edges of the box are the 25th and 75th percentiles, the whiskers extend to the most extreme data points not considered outliers, and outliers are plotted individually.

Deterministic simulations yield a velocity of ~11cm s^−1^ in both the axon with uniformly distributed Na^+^ channels, and over clustered Na^+^ channels (Figure 7). In stochastic simulations, we obtained a comparable median value. However, as was the case with the metabolic cost of APs, shortening lipid rafts or increasing the distance between them resulted in a reduction of the AP propagation velocity.

This difference can be attributed to the lowered inward ionic current. In axons, membrane current not only depolarizes the local membrane, but it also serves to drive the waveform of APs forward. A lower membrane current will result in slower depolarization of the membrane segment “ahead,” and thus in slower AP propagation. Increasing *L*, and partially compensating by increasing the density of Na^+^ channels as done in Section 3.2 reduces the metabolic cost, and accordingly the propagation velocity of APs. In the most extreme case we considered, with 0.2 μm long lipid rafts placed 8 μm apart, the median propagation velocity was ~7cm s^−1^. In this axon, stochastically simulated APs fail to propagate in 3 trials out of 20.

### 3.4. Ionic mechanisms behind the reduction of metabolic cost

**Figure 8:**
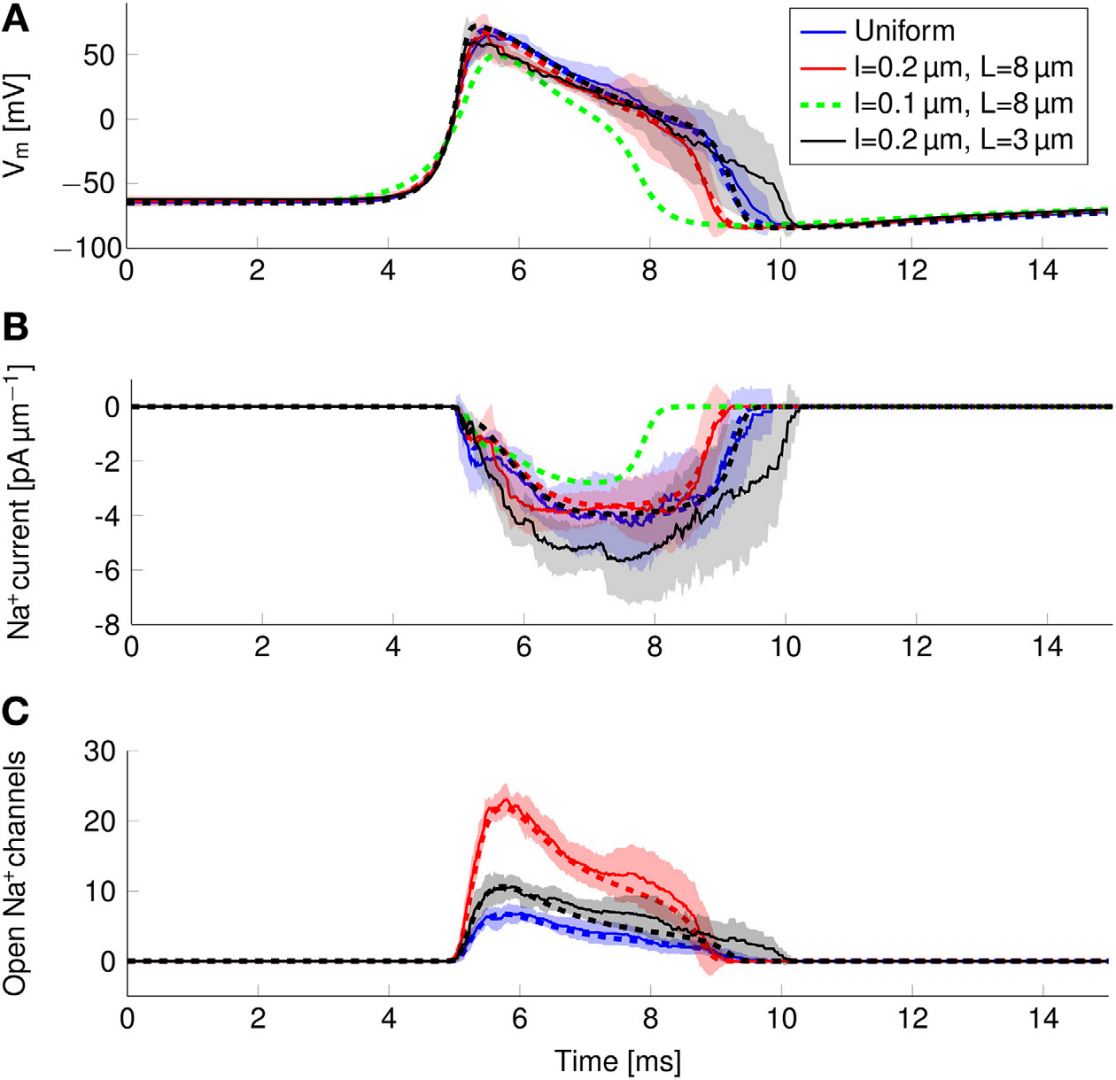
**Action potential and sodium current waveform in uniform channel density axons (Blue, STD shaded light blue, deterministic results in blue dotted line), in 0.2 μm long clusters placed 3 μm apart (Black, STD shaded gray, deterministic results in black dotted line), in 0.2 μm long clusters placed 8 μm apart (Red, STD shaded light res, deterministic results in red dotted line) and in 0.1 μm long clusters placed 8 μm apart (Dotted green line) in a 0.1 μm diameter C-fiber axon. (A)** The action potential waveforms **(B)** Instantaneous Na^+^ current and **(C)** open Na^+^ channels in a single compartment. Although there are few Na^+^ channels open in the repolarizing phase of the AP, the larger difference between *V_m_* and *E*_Na^+^_ creates a Na^+^ current comparable to that of the earlier stages.

In order to find the mechanism behind the reduction of metabolic cost, we plotted the instantaneous Na^+^ current and number of open Na^+^ channels over the course of an AP (Figure 8). In the more metabolically efficient axons (green and red lines and shades), APs are shorter (Figure 8A, red and green curves) than in the axon with uniformly distributed Na^+^ channels, or the axon with 0.2 μm long lipid rafts placed 3 μm apart.

The shortening of APs is due to a shorter period of Na^+^ current activity (Figure 8B) in metabolically efficient axons. The Na^+^ current seems to be relatively constant over the course of the AP. However, plotting the number of open Na^+^ channels (Figure 8C) reveals that Na^+^ conductance reaches its peak near the peak of the AP. In the repolarizing phase, the number of open Na^+^ channels decreases markedly due to inactivation. But approximately halfway through repolarization, Na^+^ channels can open again. This late reopening causes the “bump” in the repolarizing phase of the AP waveform. In the more metabolically efficient axons, the number of open Na^+^ channels decreases faster, the reopening is less pronounced, and the end of Na^+^ current is reached earlier. This explains the lower overall transfer of Na^+^ charge in these axons.

We can also explain the lower metabolic cost of APs in stochastic simulations compared to deterministic simulations of the same axon (Figure 6). In stochastic simulations, there is more reactivation of Na^+^ channels in the repolarizing phase as compared with deterministic simulations (Figure 8C). This effect is due to the “positive feedback” of Na^+^ channels. The random opening of any Na^+^ channel prolongs the repolarizing phase, and makes the opening of other Na^+^ channels more likely.

### 3.5. The observed effects in rafts are caused by lower Na^+^ channel density

**Figure 9:**
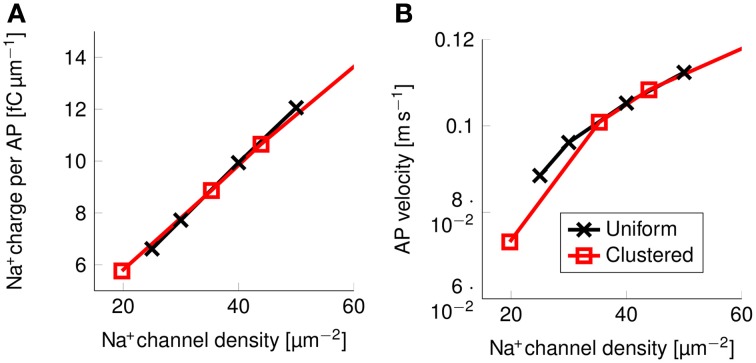
**Metabolic cost and action potential velocity as a function of Na^+^ channel density in a 0.1 μm diameter C-fiber axon. (A)** Metabolic cost and **(B)** action potential velocity as a function of Na^+^ channel density in both axons with uniformly distributed Na^+^ channels (black) and axons with clustered Na^+^ channels (red). Although the overall density of Na^+^ channels clearly has an impact on both metabolic cost and velocity, there is no detectable effect from clustering Na^+^ channels in lipid rafts.

The effect of clustering Na^+^ channels on the shape and metabolic cost of APs could simply be due to lower overall Na^+^ channel densities. In order to verify if the clustering of Na^+^ channels was in fact the responsible for the reduced metabolic cost, we simulated axons with uniformly distributed Na^+^ channels by varying the density of said channels, and plotted the resulting metabolic cost (Figure 9).

There is no noticeable difference between the metabolic cost of APs in the axon with uniformly distributed Na^+^ channels and the metabolic cost of APs propagating along an axon with Na^+^ channels clustered on lipid rafts if both axons have the same overall Na^+^ channel density (Figure 9A). Equivalently, the propagation velocity of APs is also the same in both types of axons, if the Na^+^ channel density is kept constant (Figure 9B). Our results show that the observed effect on the metabolic cost is due solely to the reduced equivalent Na^+^ channel density. That is, reducing the density of Na^+^ channels in the uniformly distributed channels model produces the same short and metabolically efficient axons than in the clustered model.

**Figure 10:**
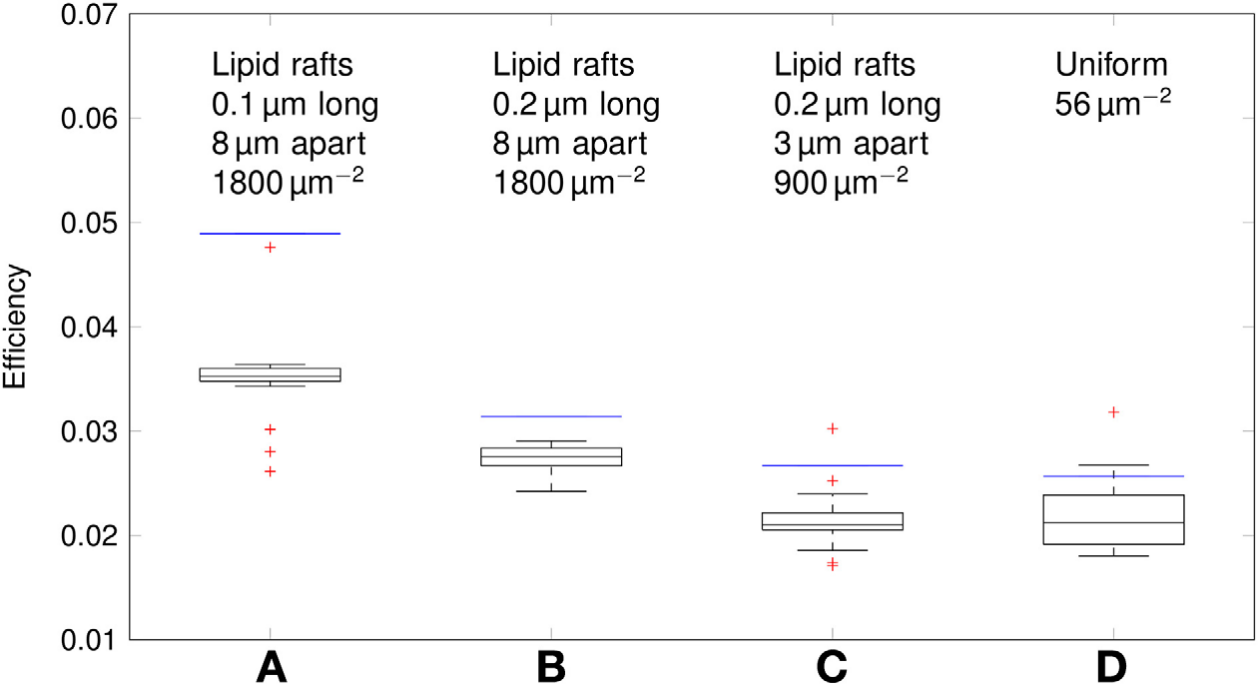
**Efficiency of action potentials for different channel distributions in a 0.1 μm diameter C-fiber axon**. Data was obtained using both deterministic (blue lines) and stochastic (boxes) simulations. **(A)** *l* = 0.1 μm, *L* = 8 μm, optimal values given by Zeng and Tang (2009), Na^+^ channel density 1800 μm^−2^, **(B)** *l* = 0.2 μm, *L* = 8 μm, Na^+^ channel density 1800 μm^−2^, **(C)** *l* = 0.2 μm, *L* = 3 μm, Na^+^ channel density 900 μm^−2^, based on data on lipid raft placement given by Pristerà et al. (2012) and personal communication with Amber Finn. **(D)** Uniform distribution. On each box, the central mark is the median, the edges of the box are the 25th and 75th percentiles, the whiskers extend to the most extreme data points not considered outliers, and outliers are plotted individually.

Using our data, we can estimate the efficiency of AP propagation in C-fiber axons. Here we define the efficiency as Na^+^ influx needed to charge membrane capacitance to AP peak/Na^+^ influx per AP. Na^+^ influx to charge membrane capacitance to AP peak is given by *dC_m_*Δ*V* where Δ*V* is the AP amplitude. The effective Na^+^ influx per AP can be estimated using our simulation data. For the 0.1 μm diameter C-fiber axon, we have plotted the results in Figure 10.

**Figure 11:**
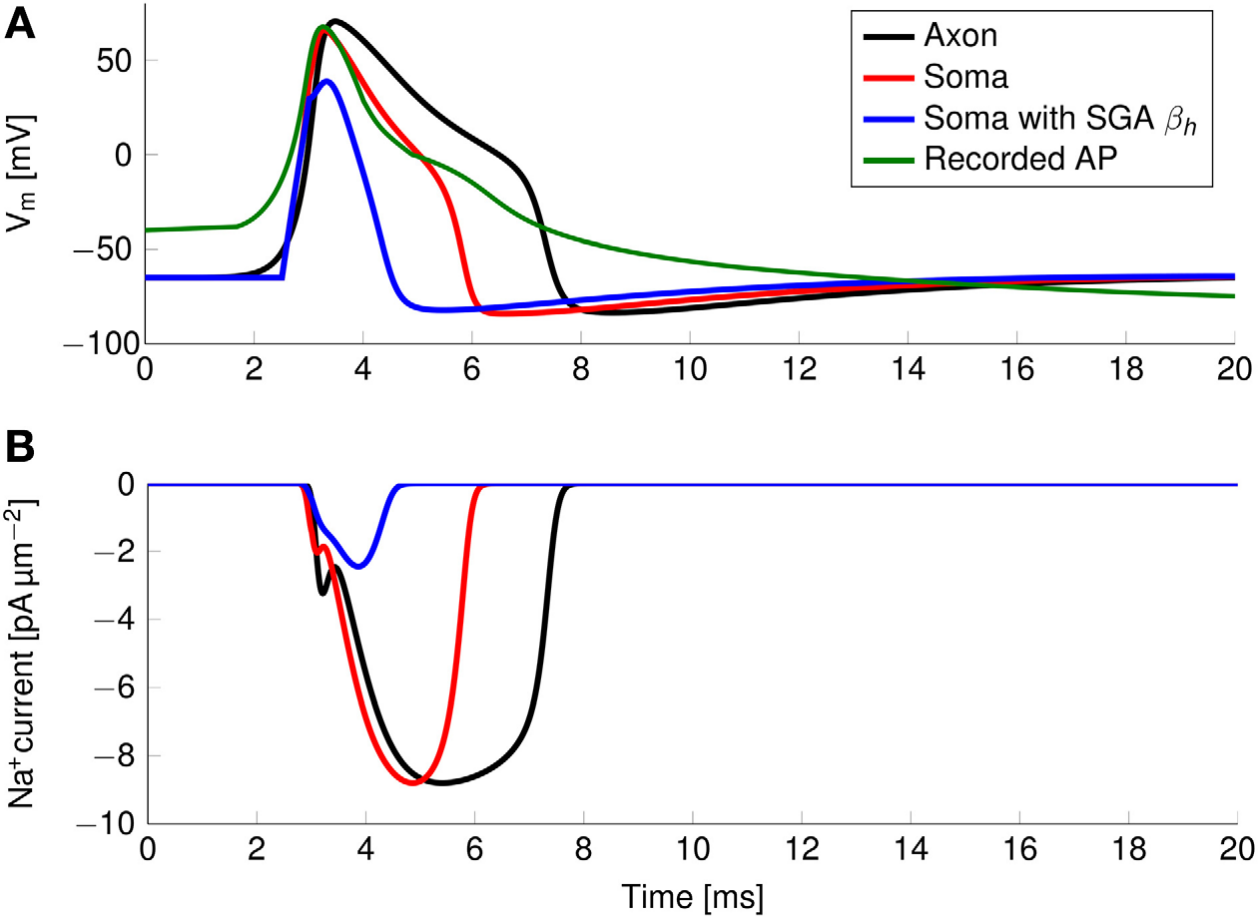
**(A)** Simulated and recorded action potentials and **(B)** sodium current waveform in a uniform channel density axon (Black) and in a soma (Red). The green curve in **(A)** is reproduced from Figure 6 in Baker (2005). The recorded AP is elicited by a long period of current injection, and therefore the membrane potential before the AP is not representative of the true resting potential, reported to be -58 mV. The AP is wider and more metabolically expensive in the axon.

**Figure 12:**
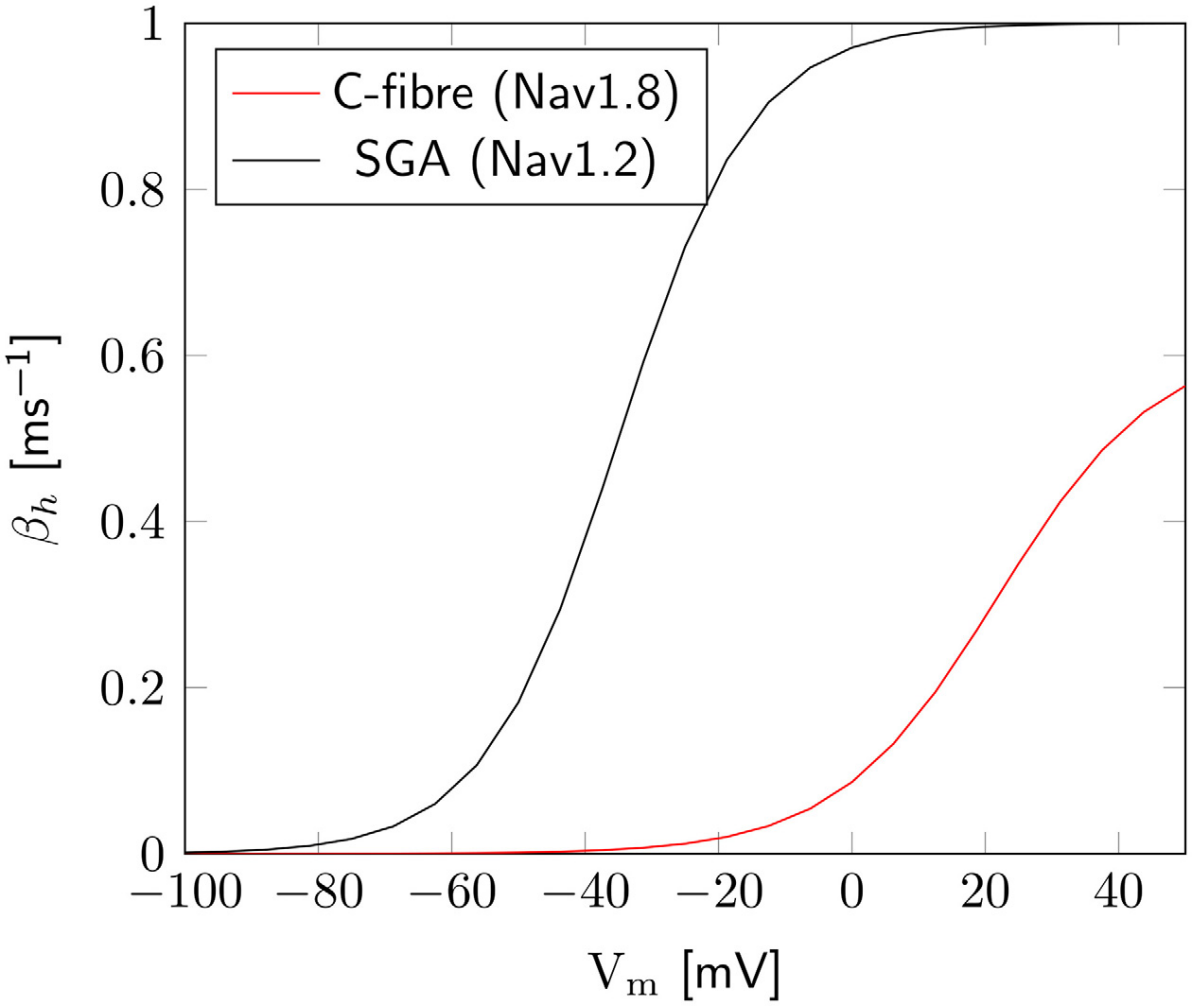
**Difference of inactivation kinetics between Nav1.2 (SGA) and Nav1.8 (C-fiber) channels**. Here we plot the β_*h*_ function, as defined in the *m*^3^*h*, or 8-state Na^+^ channel model. β^1.8^_*h*_ is much lower than β^1.2^_*h*_ over the whole biophysically relevant range.

The axon with uniformly distributed ion channels is consuming almost 50 times the capacitive minimum current necessary to charge its membrane to AP peak. The least inefficient axon in this figure still is 20 times more expensive than the theoretical minimum. To check if this inefficiency is specific to the axon, we simulated a simple spherical membrane using the same ion channel densities and physiological data than the axon. We then compare the AP waveform in the spherical compartment and the axon in Figure 11. The AP in the axon is wider than both the AP simulated in our spherical compartment, and recorded APs from DRG cells (Baker, 2005). Note that the recorded APs were elicited by a rather long current injection in the cell. In the soma, the effective Na^+^ current is ~20 times the capacitive minimum current. This inefficiency factor is much larger than even notably inefficient axons such as the squid giant axon (Hodgkin, 1975; Vetter et al., 2001). This inefficiency seems due to incomplete inactivation of Na^+^ channels. Indeed, plotting the β_*h*_ function used for Na^+^ channels in this channels reveals a significantly delayed inactivation compared to that used for Na^+^ channels in the squid giant axon, for instance (Figure 12).

In order to confirm this, we simulated a spherical membrane compartment using physiological data from the C-fiber axon model, but with the same β_*h*_ function as the squid giant axon Na^+^ channels. The shape of the AP waveform and Na^+^ current in this model is plotted in Figure 11. Action potentials in this model are much shorter than with the original kinetics for Nav1.8, and the total amount of Na^+^ current crossing the membrane is correspondingly smaller. This results in a higher efficiency for the revised kinetics model, the inefficiency factor (Effective Na^+^ current over minimum Na^+^ current) being ~3, compared to ~17 for the soma with original Nav1.8 kinetics. These calculations take into account the difference in the amplitudes of APs between the two models.

## 4. Discussion

We find that microsaltatory conduction is possible in C-fiber axons with Na^+^ channels attached to lipid rafts, i.e., action potentials (APs) can propagate from one cluster of Na^+^ channels to the next in thin C-fiber axons. We also show how late reactivation of Na^+^ channels affects the average shape of AP waveforms and increases the metabolic cost of APs in thin axons. Reducing the density of Na^+^ channels in both axons with uniformly spaced Na^+^ channels and axons with clustered Na^+^ channels results in shorter and therefore more metabolically efficient APs.

Varying the length of lipid rafts (*l*) and the distance between them (*L*) effects the shape of AP waveforms because of the associated changes in the Na^+^ channel density. Smaller rafts, as well as rafts placed further apart from each other result in smaller Na^+^ channel densitie and reduced AP width. This is due to reduced reactivation of Na^+^ channels in the repolarizing phase of the AP. Because in this phase the membrane potential is already far from Na^+^ reversal potential (E_Na^+^_), a large current, comparable to the current at the peak of the AP, crosses the membrane through any randomly reactivated Na^+^ channel.

The reactivation of even a small number of channels maintains the membrane potential in a depolarized state longer. This in turn opposes the repolarization of the membrane, leaving more time for the possible opening of other channels. This positive feedback effect makes APs slightly wider in stochastic simulations, where the possible stochastic opening of channels is taken into account. The discretization of ion channel conductances amplifies this effect, by increasing the minimum conductance. Since the effect of the opening of each channel is bigger in smaller axons (Faisal et al., 2005), reactivation of Na^+^ channels results in slightly wider action potentials in thinner axons.

Our simulations lead to two new findings regarding the metabolic cost of propagating APs in C-fibers. First, incomplete inactivation of Nav1.8 channels, the primary voltage gated Na^+^ channels in C-fibers, leads to a long lasting Na^+^ current. This in turn creates very wide APs, which are metabolically very expensive. The Na^+^ charge transfer necessary for one AP in a spherical membrane using these Na^+^ channels is 17 times more than the minimum charge transfer needed to depolarize the membrane to AP peak. This value is higher than 4, previously obtained for squid giant axon channels (Hodgkin, 1975; Attwell and Laughlin, 2001), and much higher than the very metabolically efficient channel kinetics (Alle et al., 2009; Sengupta et al., 2010). However, the latter kinetics are obtained in higher temperatures and these comparisons should only be used as an illustration.

Although incomplete inactivation has been shown to allow fast spiking (Carter and Bean, 2009), it is not clear why slow firing fibers such as C-fibers exhibit the same phenomena. C-fibers are quiet in the absence of stimulation, and their firing rate does not seem to exceed ~2 Hz (Black et al., 2004; Obreja et al., 2010). Presumably, the very slow firing rates of these high-threshold fibers reduce the impact of metabolic cost of signaling in C-fibers. The very wide APs may have a functional role by ensuring a strong post-synaptic response (Klein and Kandel, 1980; Augustine, 2001), and thus prioritize APs carried by C-fibers. Another explanation may be that incomplete deactivation plays a role in ensuring transmission of APs in noise-prone thin fibers. Nav1.8 are not the only channels expressed on C-fibers (Black et al., 2002, 2012; Vasylyev and Waxman, 2012) and there is evidence for other channel types to be present uniformly along these axons. It is possible that these channels allow for lower Nav1.8 densities. The role of the Nav1.8 channels could then be to ensure a wide action potential, and clustering them together would lower the overall metabolic cost. More detailed simulations are needed to test this hypothesis.

We also find that the cost of propagating APs in axons is significantly higher than that of an AP in a spherical membrane compartment. In our simulations, the cost of propagating action potentials in axons is roughly three times the cost estimated at the soma. The higher cost is associated with wider APs in the axon than in the soma. Our simulations use the same Na^+^ channel kinetics in the soma model and in the axon, and the broadening effect can thus only be attributed to the spatial arrangement of the membrane, as opposed to channels kinetics (Hallermann et al., 2012 for instance, use different channel kinetics in their model which leads to narrower APs in axons).

Lipid rafts play a role in organizing trafficking and localization of proteins on the membrane and the clustering of Na^+^ channels over lipid rafts may be beneficial in this context. Lipid rafts may allow colocalization of ionic pumps and Na^+^ sensitive channels with Nav1.8 channels, which may have some beneficial results on the cell's ionic homeostasis by placing ion pumps near the sources of current.

Because there is no myelin sheath around C-fiber axons, the membrane capacitance and leak conductance are too high for Na^+^ clusters to be placed at distances on the order of the axon's length constant (λ ≈ 200 μm). In our simulations, the maximum distance *L_max_* between lipid rafts which allowed action potential propagation was ~20 μm. The proximity of lipid rafts makes the waveform of the action potential virtually unchanged, compared to the waveform in an axon with uniformly distributed Na^+^ channels. This is in stark contrast with myelinated axons, where the myelin sheath lowers the capacitance and leak conductance of the membrane. As a result, nodes of Ranvier can be placed much further apart.

## Funding

Ali Neishabouri was supported by the UK Engineering and Physical Sciences Research Council (EPSRC). A. Aldo Faisal is supported by the Human Frontiers in Science Program Grant (HFSP RPG00022/2012).

### Conflict of interest statement

The authors declare that the research was conducted in the absence of any commercial or financial relationships that could be construed as a potential conflict of interest.
